# Repeatability and Reproducibility of Eight Macular Intra-Retinal Layer Thicknesses Determined by an Automated Segmentation Algorithm Using Two SD-OCT Instruments

**DOI:** 10.1371/journal.pone.0087996

**Published:** 2014-02-05

**Authors:** Xinting Liu, Meixiao Shen, Shenghai Huang, Lin Leng, Dexi Zhu, Fan Lu

**Affiliations:** School of Ophthalmology and Optometry, Wenzhou Medical University, Wenzhou, Zhejiang, China; Medical University of South Carolina, United States of America

## Abstract

**Purpose:**

To evaluate the repeatability, reproducibility, and agreement of thickness profile measurements of eight intra-retinal layers determined by an automated algorithm applied to optical coherence tomography (OCT) images from two different instruments.

**Methods:**

Twenty normal subjects (12 males, 8 females; 24 to 32 years old) were enrolled. Imaging was performed with a custom built ultra-high resolution OCT instrument (UHR-OCT, ∼3 µm resolution) and a commercial RTVue100 OCT (∼5 µm resolution) instrument. An automated algorithm was developed to segment the macular retina into eight layers and quantitate the thickness of each layer. The right eye of each subject was imaged two times by the first examiner using each instrument to assess intra-observer repeatability and once by the second examiner to assess inter-observer reproducibility. The intraclass correlation coefficient (ICC) and coefficients of repeatability and reproducibility (COR) were analyzed to evaluate the reliability.

**Results:**

The ICCs for the intra-observer repeatability and inter-observer reproducibility of both SD-OCT instruments were greater than 0.945 for the total retina and all intra-retinal layers, except the photoreceptor inner segments, which ranged from 0.051 to 0.643, and the outer segments, which ranged from 0.709 to 0.959. The CORs were less than 6.73% for the total retina and all intra-retinal layers. The total retinal thickness measured by the UHR-OCT was significantly thinner than that measured by the RTVue100. However, the ICC for agreement of the thickness profiles between UHR-OCT and RTVue OCT were greater than 0.80 except for the inner segment and outer segment layers.

**Conclusions:**

Thickness measurements of the intra-retinal layers determined by the automated algorithm are reliable when applied to images acquired by the UHR-OCT and RTVue100 instruments.

## Introduction

Evaluation of intra-retinal layer thickness plays an important role in the diagnosis and monitoring of various retinal diseases. For example, thinning of the retinal nerve fiber layer (RNFL) is often noted in glaucoma and myopia [1], [2], and thinning of the ganglion cell complex (GCC) occurs during the development of glaucoma [3]. The thickness of the outer nuclear layer (ONL) in the fovea was reported to correlate with visual acuity in central serous chorioretinopathy eyes [4]. In early age-related macular degeneration when vision field defects are present, the photoreceptor outer segment (OS) layer thins and the retinal pigment epithelium (RPE) thickens [5]. Segmentation of intra-retinal layers is very important not only in ophthalmology but also in neurology. Except for the RNFL, the thicknesses of the deeper retinal layers are reported to change in multiple sclerosis, Parkinsonian syndromes, and less frequently in disorders such as neuromyelitis optica and Wilson’s disease [6], [7], [8], [9], [10].

Optical coherence tomography (OCT) is a noninvasive, noncontact diagnostic tool that can provide in vivo cross-sectional images of the retina with high resolution [11]. It has become an essential tool for diagnosing and monitoring the development of various retinal pathologies [12], [13], [14]. Spectral domain OCT (SD-OCT) is the most readily available OCT system for retinal imaging and has a faster scan speed and higher axial resolution than time domain OCT. Currently, most of the commercially available SD-OCT instruments have a resolution of approximately 5 µm [15], [16]. Ultra-high resolution OCT (UHR-OCT), with an axial resolution of approximately 3 µm or less, has the ability to image retinal ultrastructure [17], [18], [19]. Because the segmentation software of most commercial systems is limited to measuring the thicknesses of only a few layers, such as the total retina and the RNFL, several computer automated algorithms for segmenting intra-retinal layers have been proposed to quantitatively evaluate the thickness of more layers that can be imaged with advanced SD-OCT imaging techniques [16].

The use of automated algorithms has undoubtedly enhanced the quantitative diagnosis of ophthalmic disease. To our best knowledge, however, there are few studies that test the reliability of the intra-retinal layer thickness measurements determined by automated segmentation of OCT images. Knowing the level of reliability of such measurements is very important for clinical applications. Thus, the goal of this study was to investigate the repeatability and reproducibility of thickness measurements determined by an automated segmentation algorithm applied to images of eight intra-retinal layers acquired by a custom-built UHR-OCT instrument and a commercially available RTVue100 OCT (Optovue, Fremont, CA, USA) instrument.

## Materials and Methods

### Subjects

This study followed the tenets of the Declaration of Helsinki and was approved by Ethics Committee of Wenzhou Medical University. Twenty normal subjects (12 males and 8 females, mean ± standard deviation age: 25.1±2.0 years, range: 24 to 32 years) were included, and each signed an informed consent. The inclusion criteria were as follows: no history of ocular or systemic disease, 20/20 or better visual acuity, range of refractive error between −2.00 diopter (D) and +0.50 D, no history of intraocular pressure higher than 21 mmHg, and a normal appearance of the macula.

### Instruments and Image Acquisition

Retinal OCT imaging was performed with two SD-OCT instruments configured as shown in Table 1. Briefly, the UHR-OCT used a superluminescent diode (SLD: T840; SuperLum Diodes Ltd., Moscow, Russia) [12]. For imaging the posterior segment of the eye, it was adapted onto a slit-lamp system with the installation of an ocular lens (60 D; Volk Optical, Mentor, OH, USA) on the sample arm. The field of the scan was set to approximately 15° to 20°. The power of the incident light was set to 750 µW, which is well below the safety standard, according to the American National Standards Institute (ANSI Z136.1-2000). The calibrated scan depth was 1.48 mm in air. To calibrate the scan width for the retinal imaging, a model eye (OEMI-7, Ocular Instruments, Bellevue, WA, USA), with a grid implanted on the fundus, was used. Each lattice was 1 mm in width. A horizontal B-scan was performed crossing the grid, and the pixel numbers corresponding to one lattice were acquired.

**Table 1:** Configuration of the two OCT instruments.

Technical details	UHR-OCT	RTVue 100 OCT
Axial Resolution	3 µm	5 µm
Scan Speed	24,000 Scans/s	26,000 Scans/s
Center wavelength	840 nm	840 nm
Band width	100 nm	50 nm
Scan width	8 mm	8 mm
Image Size of each B-scan	1,365 pixels (depth)×2,048 pixels (width)	640 pixels(depth)×960 pixels (width)

### Procedure

Before imaging, all eyes received an ocular examination including visual acuity testing, autorefraction, intraocular pressure, and ophthalmoscopic examination. After enrollment, all eyes were imaged without mydriasis. Two repeated measurements were performed in a short time on the same day by a single examiner (XL) using both SD-OCT instruments to test the intra-observer repeatability. The OCT imaging was also performed one time by another examiner (LL) using each OCT instrument on the same day to test inter-observer reproducibility. The order of OCT examinations was chosen randomly for each patient. During OCT imaging, the subjects were asked to move their head away from the headrest after each image acquisition, and after five minutes they were asked to reposition their head for the following measurement [20]. One author (XL), who did not know which images were taken by which observer or from which subjects, processed the images.

### Measurements of Macular Thickness of Intra-retinal Layers

**Figure 1:**
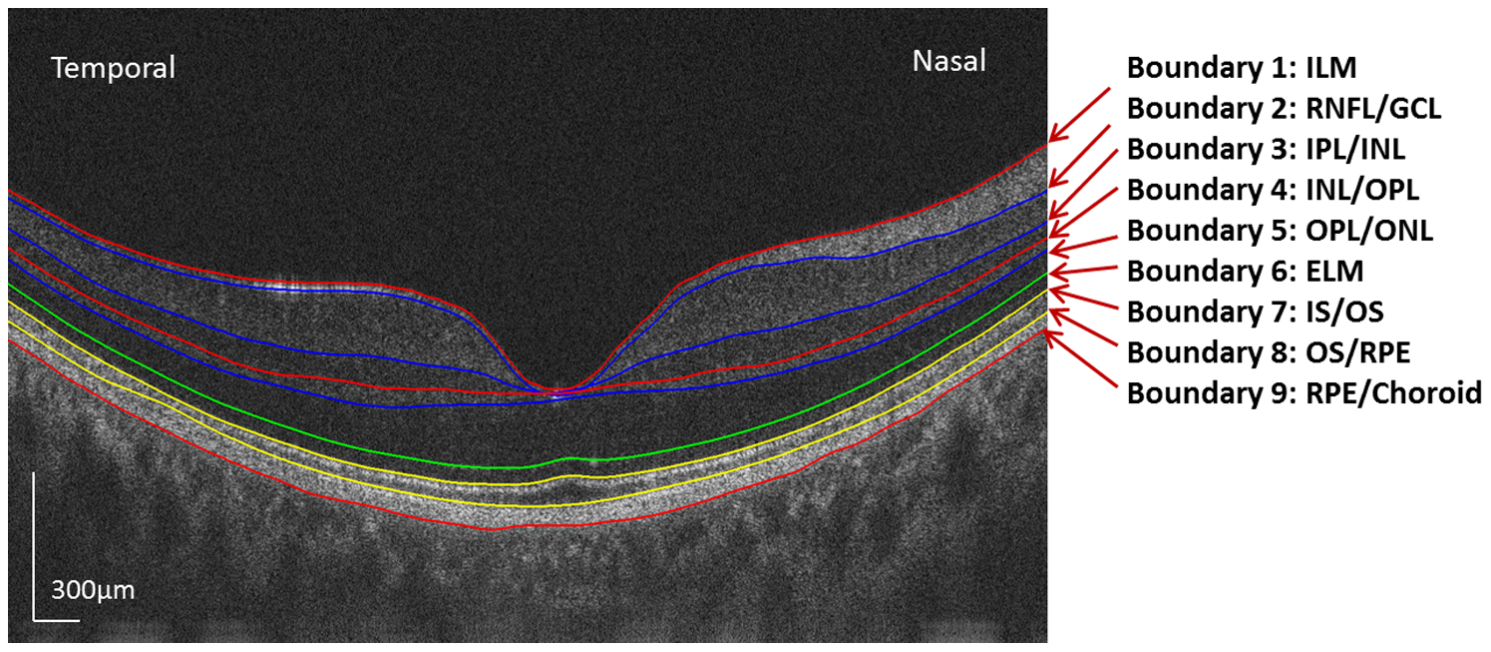
Boundaries of intra-retinal layers in OCT macular images. As seen in this image taken by UHR-OCT in the horizontal meridian, nine boundaries of intra-retinal layers were visualized. Images taken in the vertical meridian by UHR-OCT and in both meridians by the RTVue100 were similar to this.

Custom software for automatic segmentation was developed to measure the thicknesses of eight intra-retinal layers on 2-D images produced by each OCT instrument. The image segmentation algorithm mainly employed graph theory and shortest-path search based on an optimization algorithm of dynamic programming technique as described in a previous study [16]. Nine boundaries of the intra-retinal layer structures were detected (Fig. 1) including (1) internal limiting membrane (ILM); (2) nerve fiber layer/ganglion cell layer (NFL/GCL); (3) inner plexiform layer/inner nuclear layer (IPL/INL); (4) inner nuclear layer/outer plexiform layer (INL/OPL); (5) outer plexiform layer/outer nuclear layer (OPL/ONL); (6) external limiting membrane (ELM); (7) inner segment/outer segment of receptors (IS/OS); (8) outer segment/retina pigment epithelium (OS/RPE); (9) retina pigment epithelium/choroid (RPE/choroid). Each of these nine boundaries was detected sequentially by a two- step segmentation procedure. First, a graph based on node cost assignments was built. The node costs are mainly based on the intensity gradient values along the vertical direction and other features, such as the edge direction, which depended upon the boundary of interest. Second, the layer boundary was extracted by a shortest path search applied to the graph using a dynamic programming algorithm. Then, all boundaries detected were overlaid on the OCT images and were verified by visual inspection performed by one of the authors (XL). A semi-automated approach was implemented in the algorithm to correct the segmentation errors that occurred in regions which had extremely low reflectivity or almost no structural information [21], [22].

**Figure 2:**
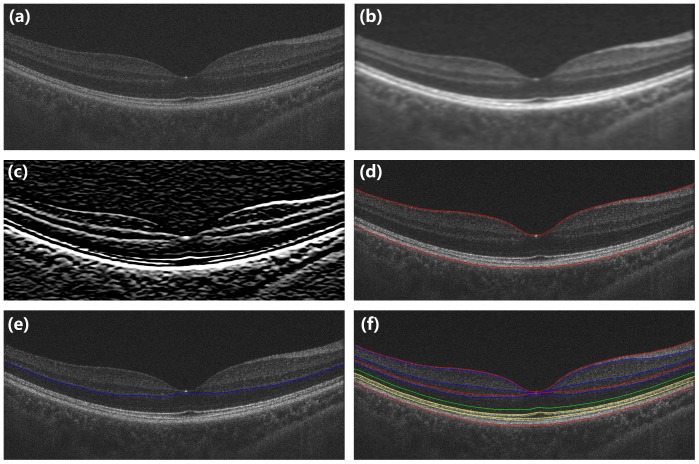
The detailed sequence in the boundary segmentation process. (a) Original image. (b) Image smoothing. (c) Gradient image. (d) The ILM and the boundary between the RPE and choroid layers were first segmented. (e) Limiting detection area and search the minimum-weighted path. (f) Segmented image.

Figure 2 illustrates the detailed sequence in the boundary segmentation process. Each OCT image was first pre-processed to reduce the background noise using median and Gaussian filtering techniques. This step helped to improve the performance of the segmentation algorithm. The ILM and the boundary between the RPE and choroid layers were first segmented so that other boundaries could be segmented in a limited search region to save computation time. The ILM was defined as the first highly reflective increase from the inner side of the retinal image. It was most often well demarcated, easily detected, and followed by a sector of high reflectivity. Based on these features, the initialized boundary was determined by the first peak on each sampling line from the inner side of the retinal structure. Then the boundary was refined by finding the shortest path in a limited region based on the initialized boundary. The RPE layer was located on the outermost side of the retina and was one of the most hyper-reflective layers within each retinal OCT image. Thus, we searched for the brightest pixel in each A-scan line below the ILM layer on the pre-processed image as an estimated boundary between the RPE and choroid layers. Then the shortest path search was applied on the graph to refine these two boundaries based on their estimated layer locations. Once these two boundaries were segmented, the process for other boundaries was repeated recursively by limiting the search space based on the previous segmentation to detect a new layer boundary.

### Statistical Analysis

All statistical analysis was performed with the Statistical Package for the Social Sciences (SPSS) software (v17.0 for Windows; SPSS Inc., Chicago, IL, USA). Descriptive statistics were determined as means ± standard deviations. The intra-observer repeatability was measured with two OCT images obtained by the same operator, and the inter-observer reproducibility was measured with two OCT images obtained by two different operators. The overall mean thickness, the coefficients of repeatability and reproducibility (COR), and intraclass correlation coefficients (ICC) were calculated to evaluate the repeatability and reproducibility of the thickness measurements. The overall mean thicknesses of the eight intra-retinal layers along the central macular 6-mm scan length were determined as the average of the first and second measurement by the same examiner. The COR was defined as the standard deviation of differences between the two measurements divided by the mean value of two different measurements. The ICC was determined based on a mixed-model analysis of variance proposed by Bartko and Carpender [23]. The paired t-test, ICC, 95% limits of agreement (LoA), and Bland and Altman plots [24] were analyzed to evaluate the agreement of thickness measurements between the two SD-OCT instruments. The 95% LoA was defined as the mean difference ±1.96 standard deviation [25]. P-values <0.05 was considered statistically significant.

## Results

**Figure 3.Thickness:**
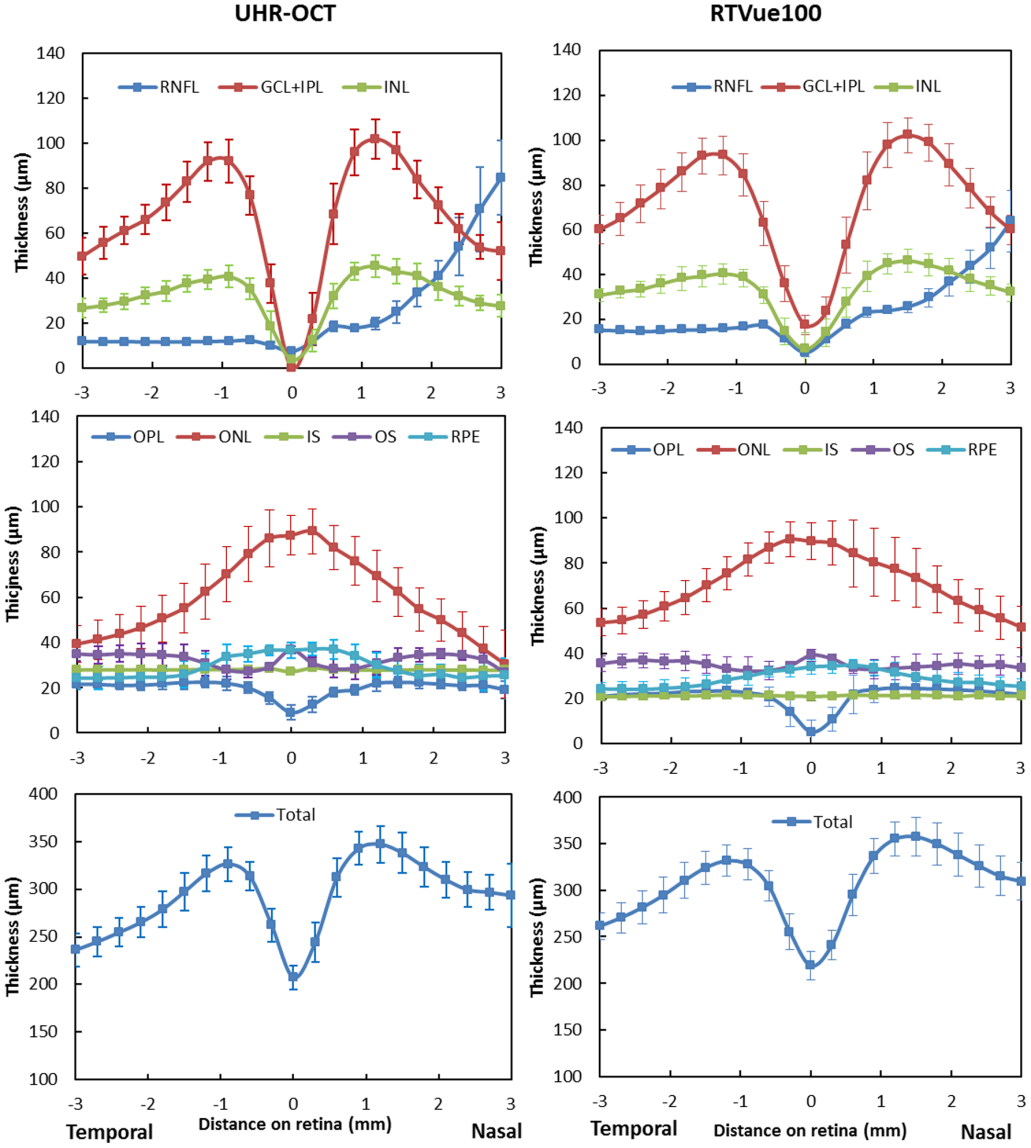
profiles of eight intra-retinal layers determined from the UHR-OCT and RTVue100 images in the horizontal meridian. Thickness profiles of eight intra-retinal layers along the horizontal meridian were averaged for 20 normal healthy eyes. Error bars represent standard deviation.

**Figure 4:**
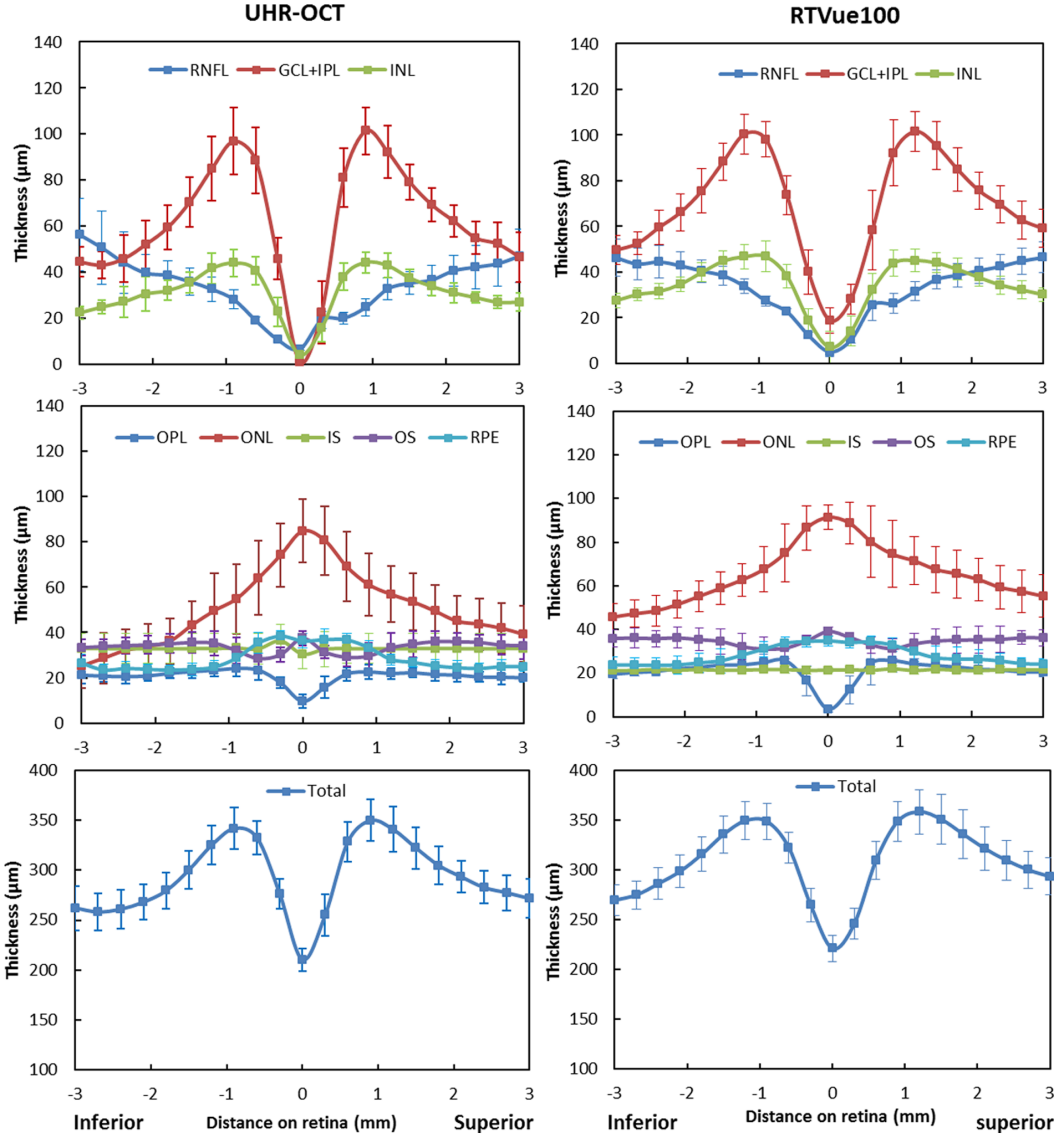
Thickness profiles of eight intra-retinal layers determined from the UHR-OCT and RTVue100 images in the vertical meridian. Thickness profiles of eight intra-retinal layers along the vertical meridian were averaged for 20 normal healthy eyes. Error bars represent standard deviation.

**Figure 5:**
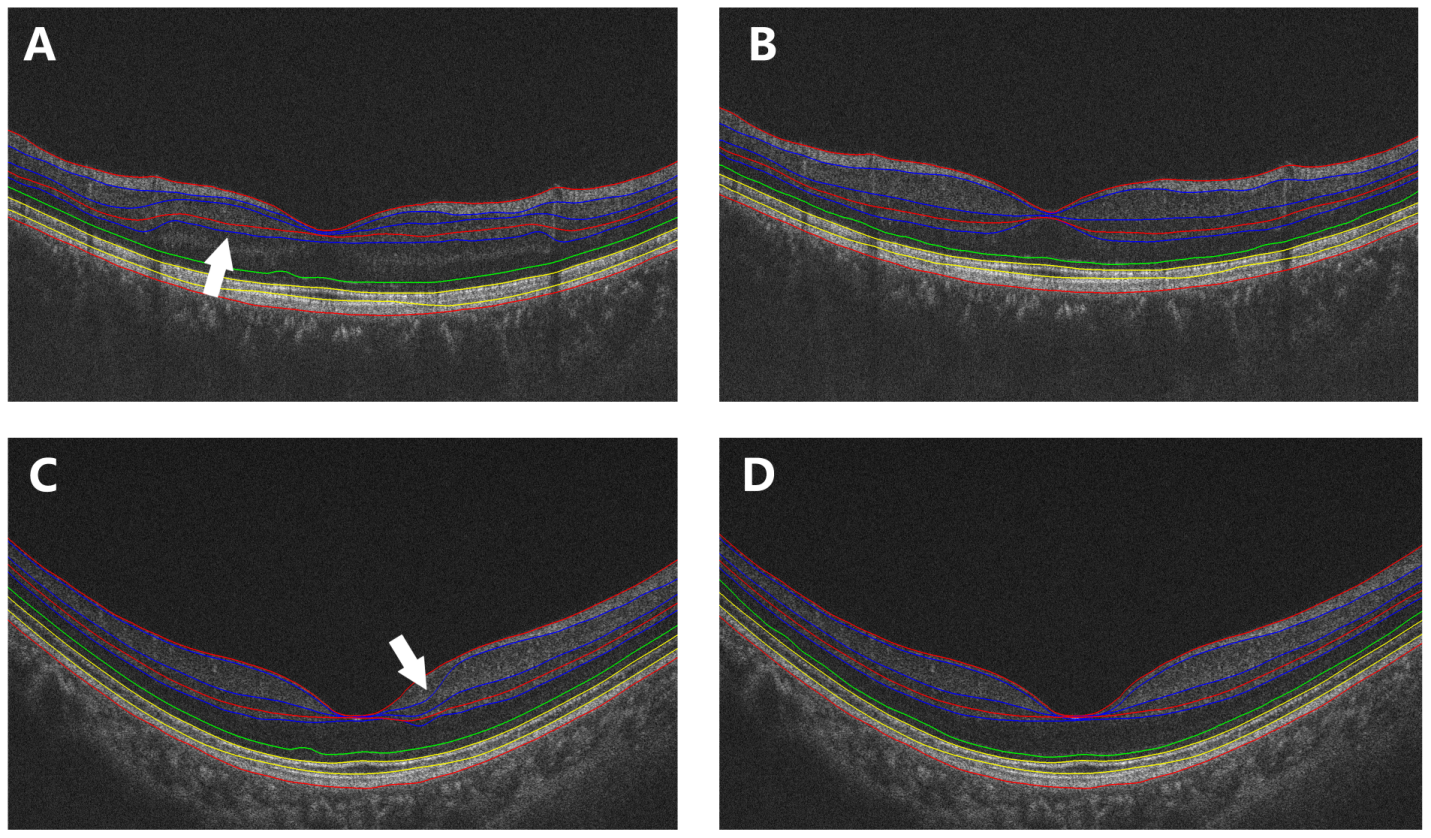
Segmentation errors in scans of lower image quality and corresponding corrected segmentation after applying the semi-automated approach. (A) The algorithm mistakenly identified the OPL/ONL interface. (B) Corrected segmentation corresponding (A) after applying the semi-automated approach. (C) The algorithm mistakenly identified the RNFL/GCL boundary. (D) Corrected segmentation corresponding (C) after applying the semi-automated approach.

The automated algorithm successfully segmented eight intra-retinal layers in the macular images obtained by the UHR-OCT and RTVue100 instruments. The algorithm also determined the thickness profiles of each layer along the 6-mm horizontal (Fig. 3) and vertical (Fig. 4) scans obtained by each instrument. There were errors in the segmentation boundary for a few images of lower quality. For example, the algorithm mistakenly identified the OPL/ONL interface in Figure 5A. A similar failure occurred for the RNFL/GCL boundary in Figure 5C. The semi-automated approach successfully corrected the segmentation errors (Fig. 5B and 5D). Visual inspection confirmed that the boundary detection for the eight intra-retinal layers was valid in all images acquired by the two OCT instruments.

For the twenty normal eyes, the overall mean total retinal thickness measured with UHR-OCT was 292.17±14.70 µm and 293.83±14.96 µm in horizontal and vertical meridians, respectively. The total retinal thickness measured with RTVue was 305.70±15.73 µm and 309.03±15.07 µm in horizontal and vertical meridians, respectively. The overall mean intra-retinal layers thicknesses ranged from 19.85 to 67.18 µm with UHR-OCT and 20.88 to 72.03 µm with the RTVue100.

Table 2 and Table 3 show the repeatability and reproducibility of thickness measurements for intra-retinal layers measured with UHR-OCT and RTVue100, respectively. There were no significant differences between the two thickness measurements for either instrument obtained by the same examiner. The ICCs obtained for the intra-observer repeatability and inter-observer reproducibility tests of both SD-OCT instruments were greater than 0.945 for the total retina and all intra-retinal layers except the IS layer, which ranged from 0.051 to 0.643, and the OS layer, which ranged from 0.709 to 0.959. The coefficients of repeatability and reproducibility were less than 6.73% for the total retina and all intra-retinal layers.

**Table 2:** Repeatability and reproducibility of thickness measurements for intra-retinal layers measured by UHR-OCT.

	T1 (µm)	T2 (µm)	T3 (µm)	T1–T2 (µm)	T1–T3 (µm)	ICC_a_	ICC_b_	COR_a_ (%)	COR_b_ (%)
**Horizontal Meridian**									
**RNFL**	22.72±2.68	22.68±2.79	22.82±2.64	0.04±0.96	−0.10±1.07	1.000	1.000	2.07	2.01
**GCL+IPL**	67.36±5.84	67.00±5.81	68.24±8.04	0.36±1.01	−0.88±5.30	1.000	0.999	1.43	2.59
**INL**	31.79±2.84	32.11±2.67	30.84±5.07	−0.32±1.18	0.95±4.53	0.997	0.995	3.20	3.80
**OPL**	19.91±1.13	19.78±1.12	19.90±1.00	0.13±1.42	0.01±0.91	0.981	0.984	4.87	4.49
**ONL**	61.71±9.17	60.49±10.24	59.56±10.45	1.23±6.41	2.15±6.46	0.998	0.995	1.98	1.85
**IS**	27.57±3.50	28.29±5.58	29.36±6.08	−0.72±5.89	−1.79±6.65	0.300	0.158	0.94	0.77
**OS**	32.05±2.35	32.21±2.11	32.61±1.78	−0.17±2.07	−0.57±2.17	0.986	0.964	2.05	2.85
**RPE**	29.06±2.50	29.59±1.90	29.28±1.82	−0.53±2.25	−0.22±1.93	0.994	0.990	1.78	3.43
**Total**	292.18±14.93	292.16±14.56	292.62±14.85	0.02±2.28	−0.44±2.04	1.000	1.000	0.36	0.50
**Vertical Meridian**									
**RNFL**	32.57±4.61	32.85±4.41	33.10±4.75	−0.28±1.25	−0.53±1.41	0.998	0.998	2.97	3.21
**GCL+IPL**	62.56±6.02	62.27±6.89	62.13±5.67	0.29±2.98	0.43±1.57	0.998	0.999	3.51	2.04
**INL**	31.49±3.09	31.43±2.37	31.87±3.10	0.06±2.18	−0.38±2.55	0.995	0.994	4.22	4.14
**OPL**	20.43±1.45	20.84±1.14	20.49±0.88	−0.41±1.33	−0.06±0.95	0.945	0.969	5.04	4.09
**ONL**	52.59±10.68	51.04±12.24	51.72±11.41	1.55±6.07	0.87±5.17	0.998	0.998	2.12	2.90
**IS**	32.22±6.77	33.60±7.60	32.46±7.68	−1.38±5.70	−0.23±4.25	0.376	0.643	1.90	2.08
**OS**	33.39±2.37	33.60±2.72	33.61±2.59	−0.21±1.28	−0.22±1.67	0.935	0.959	3.06	2.25
**RPE**	28.33±2.23	28.44±2.53	28.52±1.89	−0.11±1.02	−0.19±1.47	0.988	0.989	3.21	2.86
**Total**	293.58±14.92	294.08±15.03	293.90±14.79	−0.50±1.31	−0.31±1.23	1.000	1.000	0.40	0.46

T1: mean thickness for the first measurement by examiner 1; T2: mean thickness for the second measurement by examiner 1; T3: mean thickness for the first measurement by examiner 2; ICCa: intraclass correlation coefficients of repeatability; ICCb: intraclass correlation coefficients of reproducibility; CORa: coefficients of repeatability; CORb:coefficients of reproducibility; n = 20 eyes.

**Table 3:** Repeatability and reproducibility of thickness measurements for eight intra-retinal layers measured by the RTVue100.

	T1 (µm)	T2 (µm)	T3 (µm)	T1–T2 (µm)	T1–T3 (µm)	ICC_a_	ICC_b_	CORa(%)	CORb(%)
**Horizontal Meridian**									
**RNFL**	22.42±1.72	22.29±1.76	22.14±1.67	0.13±0.53	0.27±0.59	1.000	0.999	1.84	2.72
**GCL+IPL**	71.89±6.46	72.16±6.54	71.76±6.56	−0.27±0.82	0.14±1.00	0.999	0.999	1.74	2.15
**INL**	33.88±2.89	33.75±3.24	34.04±2.99	0.12±0.93	−0.16±1.16	0.998	0.996	2.66	3.49
**OPL**	21.08±1.63	20.68±1.60	20.37±1.10	0.40±1.75	−0.70±1.59	0.990	0.979	4.28	6.73
**ONL**	71.44±7.91	72.01±7.32	71.67±6.68	−0.58±2.73	−0.23±2.49	0.997	0.994	1.72	2.53
**IS**	21.16±1.39	21.04±1.43	21.46±1.00	0.12±0.70	−0.30±1.03	0.394	0.086	1.59	1.52
**OS**	35.43±1.90	34.43±2.45	34.64±2.02	0.99±1.89	0.79±1.88	0.889	0.935	1.93	2.24
**RPE**	28.64±2.06	29.12±1.98	29.10±1.89	−0.48±1.43	−0.46±1.34	0.987	0.971	2.39	2.60
**Total**	305.92±15.93	305.48±15.56	305.18±15.95	0.44±1.28	0.74±1.67	1.000	0.999	0.43	0.52
**Vertical Meridian**									
**RNFL**	32.75±2.92	32.64±2.76	32.75±2.92	0.11±2.08	0.00±2.02	0.999	0.999	3.54	1.71
**GCL+IPL**	69.84±5.70	69.57±5.42	69.59±5.77	0.27±1.15	0.25±1.14	0.999	0.999	1.67	1.63
**INL**	34.55±3.19	34.52±2.88	34.48±2.83	0.02±1.07	0.06±0.90	0.998	0.998	2.81	2.49
**OPL**	21.65±2.31	20.73±1.19	21.03±1.50	0.92±1.98	0.61±1.84	0.979	0.989	5.38	4.93
**ONL**	65.19±9.54	67.05±6.95	66.86±6.36	−1.86±4.06	−1.67±4.49	0.989	0.989	2.96	2.29
**IS**	21.66±1.70	21.39±1.27	21.33±1.50	0.26±1.56	0.32±2.04	0.102	0.051	1.59	1.76
**OS**	35.35±3.31	34.11±2.74	34.80±2.78	1.24±2.69	0.55±2.39	0.709	0.715	5.21	4.78
**RPE**	28.29±2.80	28.78±2.40	28.39±2.51	−0.49±1.91	−0.11±2.29	0.957	0.971	5.78	4.42
**Total**	309.26±15.47	308.79±14.71	309.25±15.02	0.47±1.82	0.01±1.01	0.999	0.999	0.56	0.65

T1: mean thickness for the first measurement by examiner 1; T2: mean thickness for the second measurement by examiner 1; T3: mean thickness for the first measurement by examiner 2; ICCa: intraclass correlation coefficients of repeatability; ICCb: intraclass correlation coefficients of reproducibility; CORa: coefficients of repeatability; CORb: coefficients of reproducibility; n = 20 eyes.

**Figure 6:**
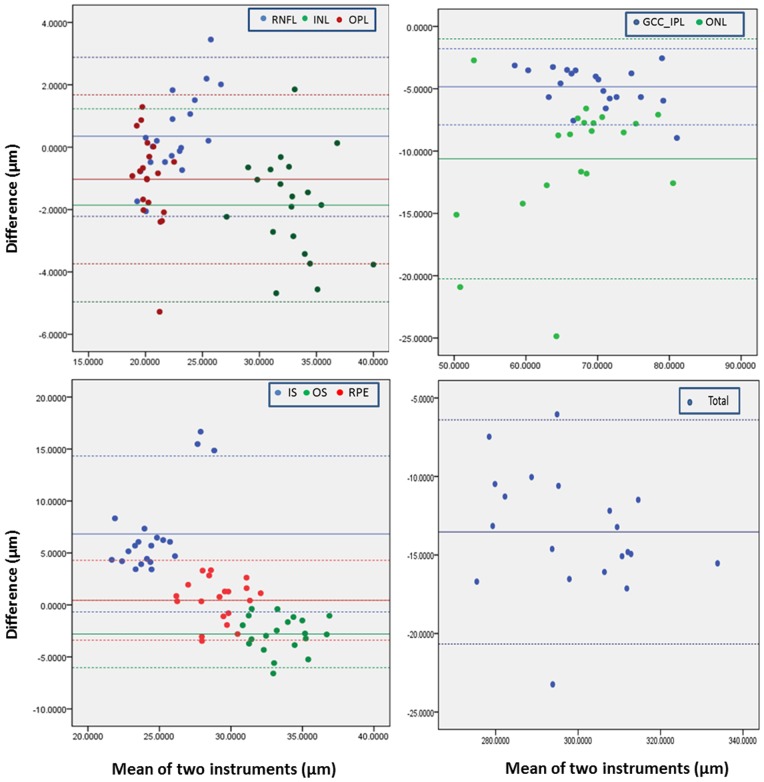
Bland-Altman plots of thickness measurements determined with the automated segmentation algorithm on UHR-OCT and RTVue100 images. Only the images along the horizontal meridian were analyzed. The horizontal full lines represent the mean of thickness differences, and the horizontal dashed lines represent the mean differences ±1.96 standard deviation.

The overall mean thicknesses of intra-retinal layers measured by UHR-OCT were compared with RTVue100 (Table 4). There were significant differences between the UHR-OCT and RTVue100 measurements of most macular intra-retinal layer thicknesses except the RNFL and RPE. The total retinal thickness measured by the UHR-OCT was significantly thinner than that measured by the RTVue100. The ICC for agreement of the thickness profiles between UHR-OCT and RTVue OCT were greater than 0.80 except for the IS and OS layers. Bland-Altman plots (Fig. 6) were also used to test the agreement of the thicknesses measured with these two SD-OCT devices. The Bland-Altman plots and the 95% LoA results showed good agreement between the two SD-OCT instruments for all of the intra-retinal layers except the ONL and IS.

**Table 4:** Comparison of the thickness measurements for eight intra-retinal layers and total retina between UHR-OCT and RTVue100 devices.

	UHR (µm)	RTVue (µm)	P value	ICC	95% LoA (µm)
**Horizontal Meridian**					
**RNFL**	22.70±2.76	22.35±1.77	0.26	0.95	−2.18 to 2.88
**GCL+IPL**	67.18±5.96	72.03±6.66	0.00	0.94	−7.89 to 1.79
**INL**	31.95±2.76	33.81±3.11	0.00	0.96	−4.96 to 1.23
**OPL**	19.85±0.90	20.88±1.39	0.01	0.94	−3.74 to 1.68
**ONL**	61.10±9.42	71.73±7.70	0.00	0.86	−20.25 to −1.00
**IS**	27.93±3.70	21.10±1.40	0.00	0.01	−0.67 to 14.33
**OS**	32.13±2.03	34.93±2.03	0.00	0.57	−6.04 to 0.44
**RPE**	29.33±1.96	28.88±1.94	0.34	0.95	−3.39 to 4.29
**Total**	292.17±15.08	305.70±16.14	0.00	0.93	−20.67 to −6.39
**Vertical Meridian**					
**RNFL**	32.71±4.59	32.69±2.71	0.96	0.98	−4.89 to 4.93
**GCL+IPL**	62.42±6.46	69.70±5.68	0.00	0.92	−13.37 to −1.20
**INL**	31.46±2.59	34.54±3.07	0.00	0.94	−5.81 to −0.34
**OPL**	20.63±1.15	21.19±1.59	0.02	0.93	−3.59 to −2.48
**ONL**	51.82±11.37	66.12±8.30	0.00	0.80	−34.20 to 5.59
**IS**	32.91±6.78	21.52±1.31	0.00	0.00	−2.17 to 24.95
**OS**	33.49±2.53	34.73±2.79	0.03	0.66	−7.34 to 4.87
**RPE**	28.39±2.39	28.53±2.49	0.29	0.96	−4.72 to 4.43
**Total**	293.83±15.35	309.03±15.46	0.00	0.90	−21.24 to −9.15

P value: tested by paired-t test.

## Discussion

It is very important in the clinical routine to know the repeatability and reproducibility of measurements. This information enables the clinician to evaluate if observed changes are due to fluctuations in the methods or if they are valid changes in the structures. This is especially true for the measurements of the intra-retinal layers, which are important morphometric parameters in the diagnosis of retinal and neurological diseases and the monitoring of the progression of these disorders. In this current study, we applied our automated algorithm to images from a custom-built UHR-OCT instrument and commercially available RTVue100 OCT instrument to yield the thickness profiles of eight intra-retinal layers. The intra-observer and inter-observer test results indicated that both instruments produced highly repeatable and reproducible measurements for most of the intra-retinal layers. The results of inter-instrument comparisons of the thickness measurements suggested that the thicknesses of intra-retinal layers obtained by the methods were not interchangeable between the two different SD-OCT instruments.

There are many studies showing the repeatability and reproducibility of the retinal measurements for normal subjects. However due to limitations of the image processing software on the commercial OCT instruments, most of them only focus on the RNFL or the total retinal thickness [15], [20], [26], [27]. The aim of our study was to report the repeatability and reproducibility of thickness measurements for eight intra-retinal layers determined by an automated algorithm applied to images from two different SD-OCT instruments. For both intra- and inter-observer comparisons, the ICCs were high for all layers except the IS. This indicates that, as in previous findings, the thickness measurements for most of the intra-retinal layers were repeatable either in different visits or by different examiners [26], [27], [28]. The repeatability of measurements in the present study is consistent with those in previous reports. Debuc et al. reported the repeatability and reproducibility of thickness measurements for six intra-retinal layers using custom-developed automated software on time domain OCT images [29]. They found ICCs to be greater than 0.75 except for the OPL and OS/RPE. Their results were consistent with ours, and the fuzzy boundaries of the ELM and OS/RPE contributed to the poor repeatability and reproducibility of IS and OS layers. The ELM was not seen clearly in most OCT images and the RPE cells of the villi were embedded into the receptor outer segments so that the OS/RPE boundary was not clear. Garas et al. reported the average and quadrant thicknesses of the RNFL and GCC based on the RTVue100 [20]. The intra-session ICC varied between 93.9% and 99.0%, intra-session CV between 1.95% and 5.69%, and intra-test variability was between 3.11 and 9.13 µm. Their results are comparable to ours. Wang et al reported the repeatability of thickness measurements for nine intra-retinal layers determined by manual segmentation of UHR-OCT images with an axial resolution of about 2 µm [30]. The ICC was >0.90 in most of the intra-retinal layers.

In the current study, we found that the repeatability and reproducibility of the UHR-OCT instrument was better than that of the RTVue100. Additionally, the repeatability of the UHR-OCT with an axial resolution below 2 µm [30] was better than that of our UHR-OCT with axial resolution of 3 µm. These results indicate that the axial resolution of OCT may contribute to the repeatability of retinal thickness measurements. This is consistent with Ge et al. [25], who reported that the higher axial resolution OCT instruments have a better repeatability in measurements of central corneal thickness and epithelial thickness. The repeatability of the IS thickness measurements in the current study was not as good as that reported by Wang et al. [30], even though they used manual segmentation in their study. Besides the axial resolution, there are some other factors, such as the image quality and image size that may also contribute to the repeatability.

The thickness measurements of most of the intra-retinal layers and the total retina were different between the UHR-OCT and RTVue100. This result was consistent with previously reported studies in which the retinal thickness measurements differed significantly depending upon the OCT systems used. For example, Seibold et al. [31] compared RNFL thickness measurements taken with three different SD-OCT instruments and a time-domain OCT instrument. RNFL thicknesses were significantly different among the four instruments, and they could not be used interchangeably. Similarly, Grover et al. found that the central subfield thickness measured by two different SD-OCT instruments differed by almost 70 µm [32]. Moreover, Wolf-Schnurrbusch et al. [33] compared central retinal thickness measurements in healthy eyes taken by six different commercially available OCT instruments. The six OCT systems each provided different results. Their results imply that the different OCT systems cannot be used interchangeably for the measurement of macular thickness.

There are some limitations to the present study. One is the unequal ratio of males to females. Setaro Oeto et al. [34] found the mean thicknesses of the INL and the OPL+ONL were significantly greater in men, and the mean RNFL thickness was greater in women. However, the purpose of our study was to evaluate the reliability of the newly developed segmentation algorithm to measure the thickness profiles of eight intra-retinal layers. Thus, gender is unlikely to influence the outcome of our study. In future studies we will pay attention to gender differences. Another limitation is that it was conducted on normal subjects only. Diseased retinal structures may vary substantially among patients, and this is likely to increase the frequency of segmentation errors. Thus the repeatability and reproducibility values may be reduced in diseased retinas [35], [36]. In future studies, we will apply our new method to a variety of retinal diseases to evaluate the clinical significance of any changes in the repeatability and reproducibility of the intra-retinal measurements.

In conclusion, thickness measurements of the intra-retinal layers have good repeatability and reproducibility when determined by the automated algorithm applied to images acquired by the UHR-OCT and RTVue100 instruments. Use of this algorithm will be helpful in the diagnosis of retinal diseases and the evaluation of disease progression.
